# Simulator development using natural language: clinician-led innovation through artificial intelligence

**DOI:** 10.1186/s41077-026-00467-2

**Published:** 2026-07-15

**Authors:** Federico Lorenzo Barra, Serena Ricci, Edoardo Moro, Jiří Travěnec, Alessandro Costa, Luca Carenzo, Giovanna Rodella, Rosanna Vaschetto

**Affiliations:** 1 Department of Anesthesiology and Intensive Care Medicine, Azienda Ospedaliero-Universitaria “Maggiore della Carità”, Novara, Italy; 2 SIMNOVA - Centro Interdipartimentale di Didattica Innovativa e di Simulazione in Medicina e Professioni Sanitarie, Università del Piemonte Orientale, Novara, Italy; 3 Department of Informatics, Bioengineering, Robotics, and Systems Engineering, University of Genova, Genova, Italy; 4 Simulation and Advanced Education Center, University of Genova, Genova, Italy; 5 Department of Anesthesiology and Intensive Care Medicine, Azienda Sanitaria Universitaria Giuliano Isontina, Trieste, Italy; 6 Department of Simulation Medicine, Faculty of Medicine, Masaryk University, Brno, Czech Republic; 7 Department of Anesthesiology and Intensive Care Medicine, Presidio Ospedaliero SS. Trinità, ASL Novara, Borgomanero, Italy; 8 Department of Anesthesia and Intensive Care Medicine, IRCCS Humanitas Research Hospital, Via Manzoni 56, Milano, Rozzano 20089 Italy; 9 DIMET - Department of Translational Medicine, Università del Piemonte Orientale, Novara, Italy

**Keywords:** Medical Education, Simulation-Based Learning, Natural Language Programming, Open Source Software, Mechanical Ventilation, Educational Technology, Healthcare Innovation, Artificial Intelligence

## Abstract

**Background:**

Healthcare simulation training faces significant barriers due to the “clinician-developer gap,” where educators lack programming expertise to create customized digital simulators. Natural Language-Driven Development (NLDD) is an emerging paradigm that enables clinicians to develop educational technology through conversational artificial intelligence interfaces.

**Methods:**

We implemented NLDD methodology to develop *Open Vent Sim*, a comprehensive mechanical ventilation simulator designed to replace anesthesia machines and ventilators in educational contexts lacking dedicated equipment. A multidisciplinary team comprising anesthesiologists, residents, a research nurse, IT, and biomedical engineers collaborated using Google AI Studio to iteratively create a web-based application through natural language prompts. Development proceeded through conversational cycles in which clinical requirements were translated into functional code via large language model assistance.

**Results:**

*Open Vent Sim* was successfully developed in about 40 h over two weeks, featuring three simulation environments: anesthesia workstation, ICU ventilator, and high-flow oxygenation systems. The simulator incorporates physiological patient profiles (normal, ARDS, COPD) with dynamic compliance calculations and realistic waveform generation. Clinical validation was achieved through the integration of continuous resident feedback during iterative development. The application was successfully implemented in SimZone 1 as an interactive skill trainer and in SimZone 2 for team-based clinical scenarios during formal anesthesia and critical care education. Significant technical adaptation was required to transform the AI-generated prototype into a production-ready application.

**Conclusions:**

NLDD demonstrates the potential to democratize the creation of educational technology by empowering clinical domain experts to develop sophisticated simulation tools without traditional programming expertise. This approach addresses resource limitations while maintaining clinical authenticity, though professional technical oversight remains essential for production-ready implementations.

**Supplementary Information:**

The online version contains supplementary material available at https://doi.org/10.1186/s41077-026-00467-2.

## Introduction

Simulation-based training has gained widespread adoption in healthcare education as an evidence-based tool to improve provider performance, clinical processes, and patient outcomes. [1, 2] Digital simulation supports healthcare training by replicating clinical environments and equipment with high fidelity. Several studies describe three-dimensional modeling, digital twins, and high-fidelity rendering that recreate hospital wards, surgical suites, and specialized devices, while others integrate mannequins or haptic feedback to enhance tactile realism. [3, 4].

Despite proven benefits, a persistent “clinician-developer gap” exists: clinical educators often lack programming skills to create or customize digital simulators, leaving them dependent on commercial products that are expensive and not easily adaptable to local needs due to language, cultural, and organizational barriers. [5] Moreover, software systems are closed without flexibility, limiting institutions’ ability to address specific training objectives or resource constraints. [6, 7] This gap is particularly pronounced in simulation centers with limited resources, where the absence of dedicated anesthesia machines and the constraint of single-unit commercial ventilation simulators significantly restrict training capacity and flexibility.

Artificial intelligence is positioned to transform healthcare and is increasingly explored in medical education. [8, 9] However, making AI-driven development accessible to front-line clinicians remains challenging. Most physicians are not trained in software development, while AI and information technology experts lack medical domain knowledge, creating a bidirectional knowledge gap. [10] This challenge has sparked interest in “no-code” or “low-code” approaches that empower domain experts to build solutions without traditional programming. [11, 12].

The term “Vibe Coding,” introduced by computer scientist Andrej Karpathy in February 2025 [13], describes relaxed and intuitive programming with AI assistance. Instead of writing detailed code manually, developers use natural language prompts and explore AI-generated code versions through trial and error. We adopt the more formal designation “Natural Language-Driven Development (NLDD),” representing a specific implementation approach within the broader Natural Language-Oriented Programming (NLOP) paradigm. NLOP offers a transformative approach to software development by integrating generative AI with natural language processing to enable programming through natural language, thereby democratizing software creation. [14].

This paradigm evolved from Language-Oriented Programming concepts focused on creating domain-specific languages for specific problem domains. [15] Unlike traditional approaches that require deep programming expertise, NLOP leverages the universality of natural human language through AI interpretation and code generation. [14, 16, 17] Current literature on natural language programming applications in healthcare simulation remains limited, encompassing only three documented studies demonstrating early-stage implementations. [18–20] These studies reveal consistent themes, including enhanced user interaction through natural language processing, empowerment of non-programmers through accessible development tools, and emphasis on scalability in clinical education.

This proof-of-concept study aimed to develop and evaluate the feasibility, physiological fidelity, and usability of a high-fidelity, open-source mechanical ventilation simulator created through clinician-led NLDD methodology. The intended use case is team-based simulation in SimZone 2 and skill training in centers lacking dedicated anesthesia machines or ventilation simulators; the clinical and educational validity of the teaching subsequently delivered with the tool is a downstream question beyond the scope of this development report. Specific objectives were: (1) addressing local resource limitations through cost-effective simulator development; (2) achieving and verifying sufficient physiological fidelity and usability of the tool through iterative formative review by clinical experts and a validated user-experience instrument, rather than measuring learner knowledge gains, which are deferred to a planned formal study; and (3) documenting a replicable framework enabling other educators to implement this development process.

## Methods

### Study design and setting

This innovation development study was conducted at two Italian simulation centers (University of Eastern Piedmont and University of Trieste) between February and June 2025. The project employed user-centered, iterative design principles guided by clinical educational needs and resource constraints. The simulator was specifically designed to address the absence of anesthesia machines in both centers’ simulation environments and to overcome the limitation of having only a single commercial ventilation simulator software available, which created bottlenecks in educational delivery. This was a proof-of-concept innovation-development study in which the unit of evaluation was the simulator itself, its physiological fidelity, functionality, and usability. Residents and clinicians participated as domain reviewers in a user-centered, iterative design process rather than as a study sample to measure learning outcomes; accordingly, the study was not designed or powered as an educational efficacy trial.

### Team composition and development platform

The multidisciplinary development team comprised one lead anesthesiology/intensive care physician, three anesthesiologists, ten anesthesiology and intensive care residents providing clinical feedback, one intensive care research nurse, and one biomedical engineer. Technical review and code optimization were subsequently conducted by software engineering collaborators at Masaryk University, the Czech Republic. This collaborative structure reflects recommended best practices, incorporating both clinical expertise and technical specialists. [21] At the end of the iterative development phase, the resulting prototype was evaluated with the User Experience Questionnaire (UEQ) by ten members of this group, seven residents and three attending physicians, who had served as the principal learner and clinical-review cohort.

**Fig. 1:**
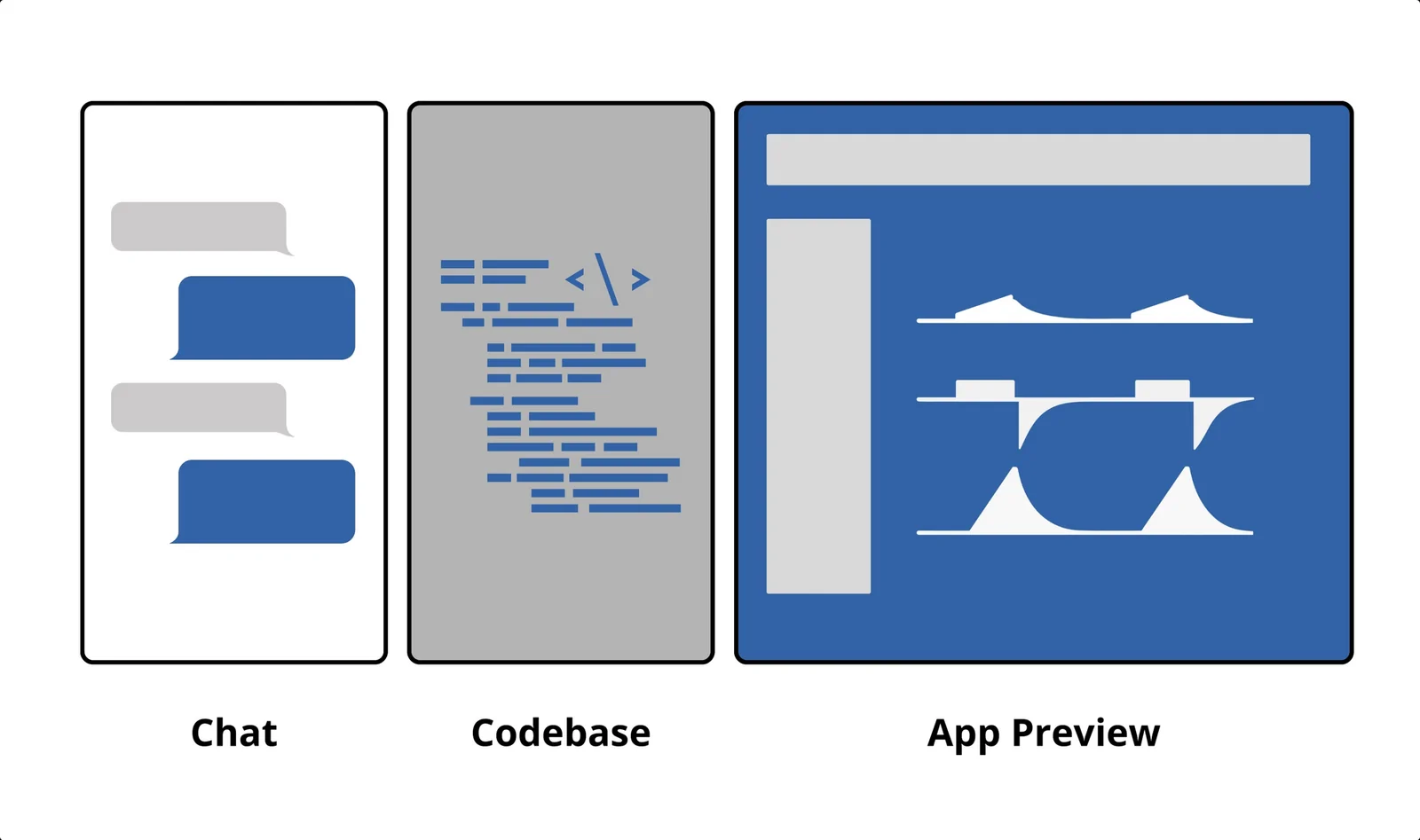
Example of development environment used for creating Open Vent Sim through the NLDD method. The interface demonstrates the three-panel synchronized configuration: the chat panel for natural language instruction input (left), the code editor on the left where the AI generates and modifies React TypeScript code (center), and the live preview panel that immediately renders the application (right)

Development used Google AI Studio (Google LLC, USA), an integrated development environment that enables natural-language interaction with large language models for real-time code generation and execution. The “Build” interface features three synchronized panels: a conversational chat window, a live code editor, and an instant application preview, enabling immediate feedback loops in which clinical prompts generate corresponding code changes with real-time visualization [Fig. 1]. The development process employed distinct tools at each phase. During initial prototyping (weeks 1–2), the clinical lead used Google AI Studio’s Build interface with Gemini 2.5 Pro as the primary development environment, generating React TypeScript code through conversational prompts. Version control was managed through Google’s integrated GitHub plugin, enabling systematic tracking of code iterations and the ability to roll back when AI-generated modifications introduced regressions. For clinical validation, evolving prototypes were deployed on Google Cloud Run to generate shareable URLs, enabling residents and faculty at both simulation centers to access and test the simulator in standard web browsers without installing local software. During the code quality evaluation phase, technical facilitators at Masaryk University worked directly with the GitHub repository using conventional development tools (Visual Studio Code, standard TypeScript tooling) to evaluate future effort necessary to refactor the AI-generated codebase into modular components, which is the expected code structure required to maintain code quality in a real-world product. Communication between clinical and technical team members was conducted through structured written feedback documents and video calls, with clinical observations documented in shared spreadsheets tracking identified issues, proposed solutions, and resolution status across development iterations.

### NLDD methodology implementation

The conceptual rationale for NLDD was empowering clinical experts to directly translate domain knowledge into functional software, bypassing traditional coding barriers. [22] Development began with comprehensive upfront planning, including the definition of educational objectives, the specification of target learning outcomes, and the documentation of detailed design requirements.

The NLDD process initiated with the foundational prompt: “Create a new web application for a ventilator simulator with basic controls and waveform displays.” Development proceeded through systematic refinement cycles in which clinical team members examined live application outputs and provided natural-language feedback ranging from high-level feature requests to granular technical specifications. The AI system interpreted multimodal inputs, including uploaded screenshots and curves’ samples of actual ventilator displays for pattern replication.

**Fig. 2:**
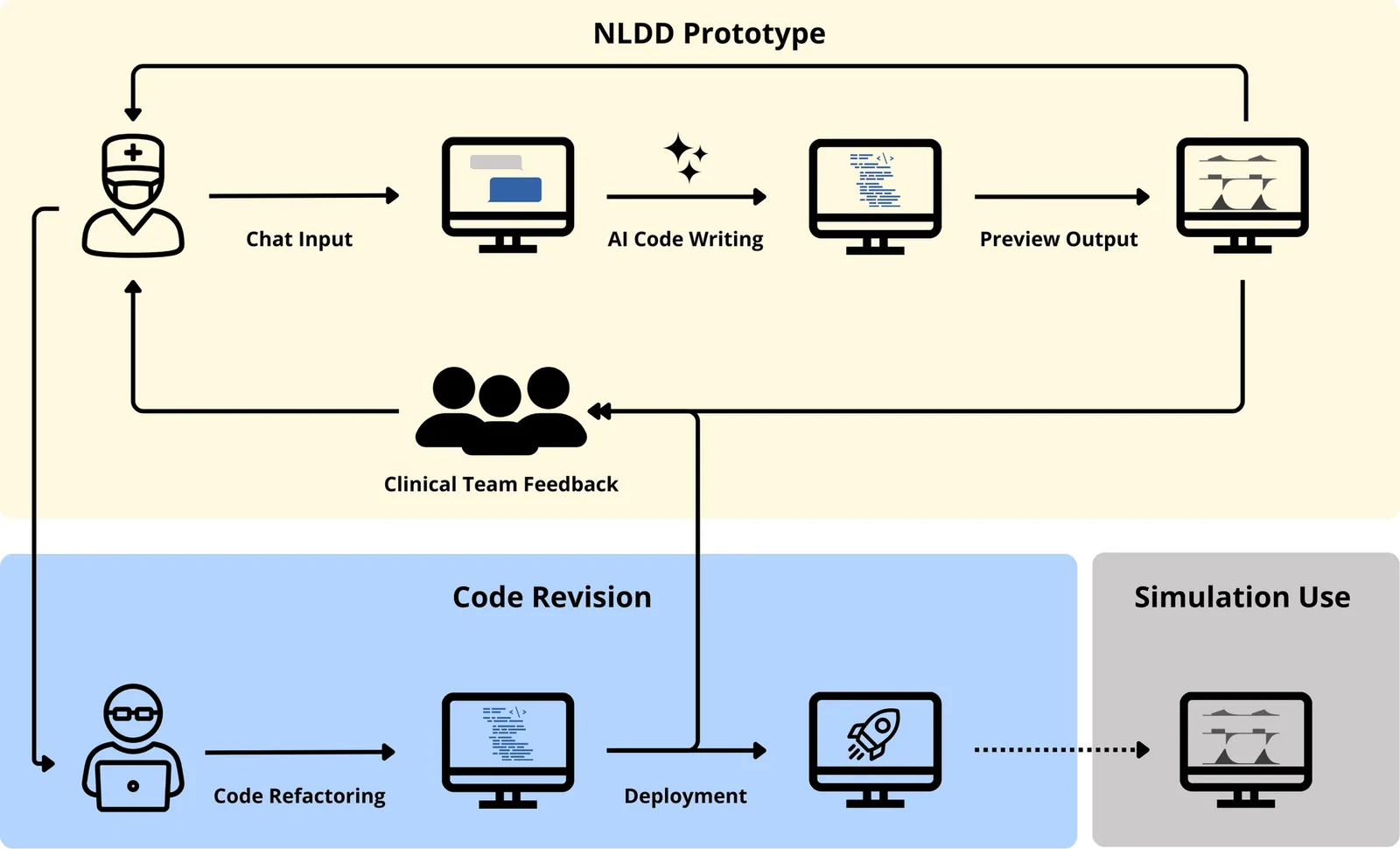
This figure outlines the development process, which is structured into three distinct phases: NLDD Prototype Phase: Clinical experts chat with generative AI development platform. The AI then writes the codebase and produces an immediate “Preview Output” of the web application. This allows for a rapid, iterative cycle where both the clinical lead and a clinical team provide feedback, which is used to refine the prototype through subsequent chat inputs. Code Revision Phase: This phase involves technical experts to ensure software quality. A developer performs code refactoring on the AI-generated code to improve its structure, remove redundancies, and enhance maintainability. After verifying the changes, the refined application is prepared for deployment. Simulation Use Phase: In this final phase, the web application becomes available for its intended purpose as a functional tool for Healthcare Simulation

Each development cycle followed a structured protocol: (1) clinical requirement articulation in natural language; (2) AI interpretation and proposed code modifications; (3) immediate implementation with live preview; (4) clinical validation and refinement specification; (5) iterative improvement until satisfactory functionality achievement. This process continued until team consensus on clinical realism and educational utility [Fig. 2].

To illustrate this iterative process, we present one representative feedback loop in detail. During the development of the Pressure Support Ventilation (PSV) mode, anaesthesiology residents identified that the initial AI-generated waveforms did not accurately depict patient inspiratory effort triggering. Specifically, the flow-time curve lacked the characteristic negative deflection preceding mechanical breath delivery that clinicians use to assess patient-ventilator synchrony. The clinical lead articulated this requirement to the AI platform as: “The PSV waveform must show a small negative deflection in the flow tracing before the ventilator delivers the breath, representing the patient’s inspiratory effort triggering the ventilator.” The AI generated a first iteration that displayed the deflection, but with unrealistic amplitude and timing. The team then refined the prompt with quantitative specifications: “The trigger deflection should be approximately − 2 to − 5 cmH2O below baseline, lasting 100–200 milliseconds before positive pressure delivery begins.” After three such refinement cycles within a single 90-minute session, the waveform display achieved clinical accuracy as confirmed by two attending intensivists. Similar feedback loops were conducted for each ventilation mode and pathophysiological profile, with clinical experts systematically reviewing waveform morphology, numerical parameter ranges, and alarm threshold behaviors against established reference standards.

### Technical implementation and validation

**Fig. 3:**
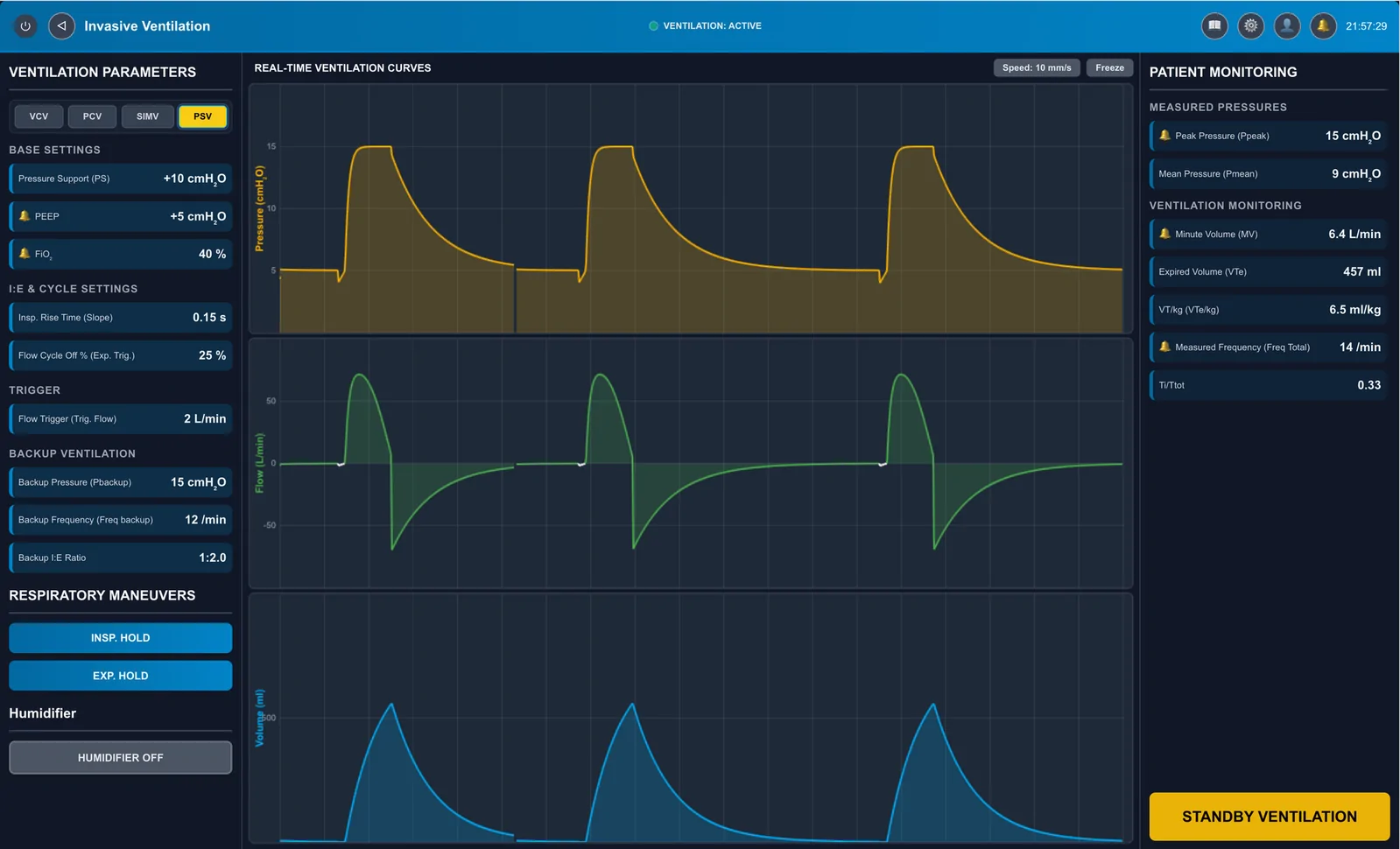
Screenshot of the complete Open Vent Sim interface illustrating the advanced functionalities of the simulator. Invasive Ventilation mode with advanced ICU interface, displaying realistic physiological waveforms and controls for respiratory maneuvers and humidifier

*Open Vent Sim* was developed featuring three primary simulation environments: anesthesia workstation, advanced ICU ventilator supporting invasive and non-invasive modalities, and high-flow oxygen delivery systems. The physiological engine incorporates pre-configured patient profiles (Normal, ARDS, COPD) and dynamic compliance calculations that affect waveform characteristics and ventilatory parameters [Fig. 3].

In terms of instructional scope, the tool is designed to let learners recognize and operate the principal ventilation modes; read and interpret pressure-, flow-, and volume-time waveforms; observe how changes in lung compliance and airway resistance alter those tracings; manage fresh-gas flow and soda-lime consumption during low-flow anesthesia; and interpret derived respiratory parameters (driving pressure, mechanical power, and respiratory-system compliance). These define what the simulator is built to support; they describe the tool’s functional and teaching capabilities and are distinct from any measured learning outcome.

The development process revealed several critical areas requiring significant technical refinement beyond the initial AI-generated code. During the early prototyping phase, the team discovered that while the AI could rapidly generate functional code for basic ventilator controls and display elements, achieving clinical-grade physiological accuracy demanded extensive manual refinement. The initial AI-generated algorithms for respiratory mechanics provided a solid foundation but required substantial modification to accurately reflect pathophysiological states. These modifications included real-time pressure-volume relationship calculations that responded appropriately to ventilator setting changes.

As the simulator evolved through user feedback cycles, the need for realistic calculations of anesthesia gas consumption became apparent. This requirement emerged from clinical educators who emphasized the importance of teaching residents about resource management and cost considerations in anesthesia practice. Therefore, sophisticated algorithms were implemented to account for multiple variables, including fresh gas flow rates, breathing circuit volume, and patient metabolic CO2 production. The soda lime absorption modeling proved particularly complex, requiring algorithms based on canister capacity and efficiency curves that responded dynamically to minute ventilation and CO2 production rates, providing realistic feedback on when canister replacement would be necessary.

### Technical adaptation and code refactoring

The technical team’s adaptation efforts extended far beyond simple bug fixes, requiring comprehensive architectural restructuring of the AI-generated codebase. The initial NLDD prototype, while functional, exhibited characteristic issues of AI-generated code, including extensive repetition and poor modularity. A significant focus was placed on implementing the “Don’t Repeat Yourself” (DRY) principle throughout the application. This architectural change ensured that modifications to parameters or visual elements required changes in only one location, dramatically improving maintainability.

Repetitive UI patterns were encapsulated into reusable React components that could be invoked with simple properties, reducing code duplication while ensuring visual and functional consistency across the application. Complex calculations and data-parsing logic were extracted into dedicated helper functions and services. This modular approach separated concerns, making the main application component cleaner while improving testability and reusability of individual functions.

The final application architecture represents a modern, type-safe, component-based system built with React and TypeScript. The configuration-driven design allows instructors to create specific clinical scenarios through a comprehensive settings panel, where they can configure anesthesia machine parameters, define patient physiological profiles by adjusting lung compliance and airway resistance, and select from pre-configured pathological presets. Advanced physiological factors such as inspiratory effort strength and expiratory flow limitation can be fine-tuned to realistically alter ventilation waveform characteristics, providing a robust platform for customized educational experiences.

Throughout development, the simulator underwent formative fidelity and content review rather than summative testing. At each iteration, residents and attending physicians examined the simulator against clinical reference standards and against screenshots and tracings of real ventilator and anesthesia-workstation displays, systematically reviewing waveform morphology, the plausibility of numerical parameter ranges, and alarm-threshold behavior. Discrepancies were resolved by consensus, requiring agreement from at least two attending intensivists before a feature was accepted (as illustrated by the pressure-support trigger example above). Each issue and its resolution were logged in a structured Google Form capturing, for every item, the screen involved, the problem detected, and the proposed solution, complemented by a shared spreadsheet tracking issue status across iterations. At the conclusion of the iterative phase, the overall usability and user experience of the final prototype were assessed with the validated Italian version of the User Experience Questionnaire (UEQ; 26 bipolar items across six scales: Attractiveness, Perspicuity, Efficiency, Dependability, Stimulation, and Novelty), completed by the learner and clinical-review cohort (*n* = 10). Responses were transformed to the standard − 3 to + 3 metric with negatively poled items reverse-scored, and scale means were computed; given the formative purpose and the convenience sample, results were analyzed descriptively (means, standard deviations, and 95% confidence intervals) and compared with the published UEQ benchmark dataset. A video example of the functioning simulator is available in the supplementary electronic files of this paper.

## Results

### Development timeline and resource requirements

A functional Open Vent Sim prototype was completed in approximately 20 h over two weeks, using iterative 1-2-hour evening development sessions interspersed with the collection of clinical team feedback. An additional 20 h of refinement achieved sophisticated feature integration and optimization of clinical realism, totaling 40 h of focused clinical expert time with minimal infrastructure costs, using free cloud-based platforms.

### Simulator features and technical challenges

The completed simulator encompasses three distinct clinical environments with comprehensive physiological modeling. Patient profile integration dynamically modifies lung compliance and airway resistance, generating realistic physiological responses to ventilator settings changes. Waveform displays accurately represent pressure, flow, and volume curves characteristic of specific pathological conditions with real-time calculations responding immediately to parameter modifications.

Later development iterations revealed inherent challenges in AI-assisted development. The primary issue was not catastrophic errors but rather the characteristic verbosity of large language model-generated code. AI models consistently produced elongated, repetitive code structures that violated fundamental software engineering principles, notably the “Don’t Repeat Yourself” (DRY) principle, ultimately resulting in a monolithic file approaching 2,300 lines, resembling the notorious “spaghetti code” phenomenon. [23].

While AI could produce functional code for almost any requested feature, code quality and maintainability were variable. This is a known caveat of AI-generated programming. [24] This pattern underscores a crucial duality: non-programmers can create functional prototypes through NLDD, but professional developers remain indispensable as consultants and quality guardians. Technical facilitators performed systematic refactoring, including breaking code into smaller components, renaming variables, simplifying logic, and removing redundant fragments. [25] The refactoring process required approximately 15 additional hours of technical specialist time to transform the working prototype into a maintainable, production-ready application with proper architectural patterns and code organization.

### Implementation and educational integration

The simulator was successfully implemented across multiple educational contexts, addressing specific resource limitations and pedagogical needs in both simulation centers.

SimZone 1 Implementation: The ventilator and anesthesia machine simulators were deployed as interactive skill trainers in SimZone 1, [26, 27] serving as hands-on learning stations following theoretical explanations of ventilation methodologies. This implementation followed established simulation pedagogy principles that emphasize the importance of bridging theoretical knowledge with practical application through structured skill practice. [28] Students could manipulate ventilator settings and observe immediate physiological responses, reinforcing theoretical concepts through interactive exploration, and transferring theoretical knowledge into applied understanding. Faculty educators reported that Open Vent Sim enabled the structured teaching of concepts previously limited by equipment unavailability, specifically anesthesia workstation ventilation modes and high-flow oxygen therapy settings.

SimZone 2 Implementation: In SimZone 2, [26, 27] the simulators were integrated into team-based clinical scenarios during formal anesthesia and critical care education sessions. This application supported small-group learning through clinical cases that required collaborative decision-making and real-time problem-solving. Facilitators observed that teams using the simulator engaged in clinically appropriate discussions, suggesting that the simulator provided sufficient fidelity to support meaningful clinical reasoning exercises. The multi-user capability allowed simultaneous training sessions, significantly increasing educational throughput while maintaining the quality of interactive learning experiences.

Specialized Procedure Training: *Open Vent Sim* supports training scenarios for complex procedures such as tracheostomy, where mechanical ventilators are unavailable. The simulator allows trainees to observe changes in ventilatory parameters and waveform alterations during simulated procedures, providing realistic feedback on airway management techniques.

Trainees and educators provided continuous formative feedback throughout development, and the usability and user experience of the final prototype were assessed with the validated UEQ (reported below). Consistent with the proof-of-concept aims of this phase, no pre-post assessment of learner knowledge was administered. A systematic evaluation of learning outcomes using validated knowledge measures represents a necessary next step and is planned as a formal multicentre study.

### User feedback integration and iterative improvements

Continuous feedback from residents, faculty, and the research nurse during implementation led to significant functional enhancements. Initial user testing revealed navigation challenges, prompting the redesign of control panels with intuitive grouping of related parameters and an improved visual hierarchy aligned with clinical workflow patterns. Clinical feedback identified discrepancies in pressure-volume relationships for pathological states, prompting refinement of compliance algorithms and addition of recruitment/derecruitment modeling for ARDS scenarios. Users requested additional monitoring capabilities, prompting the implementation of derived parameters, including driving pressure, mechanical power, and respiratory system compliance, to align with current clinical practice standards.

### Formative fidelity review outcomes

Iterative formative review progressively improved physiological fidelity, interface usability, and alignment with the intended teaching content. Early iterations were judged inaccurate on specific points, for example, the pressure–volume behavior of pathological states and the patient-trigger morphology of pressure-support ventilation, and were corrected across successive cycles until reviewers reached consensus that waveform morphology, parameter ranges, and alarm behavior matched clinical reference standards. The final prototype was confirmed by residents and attending physicians to reproduce the essential ventilator functions, realistic physiological responses, and an intuitive interface required for the targeted basic mechanical-ventilation content.

### User Experience Evaluation (UEQ)

At the end of iterative development, ten participants (seven residents and three attending physicians) completed the UEQ. All six scales were positive (i.e., above the + 0.8 threshold conventionally used to denote a positive evaluation on the − 3 to + 3 metric): Attractiveness 1.68 (SD 0.73), Perspicuity 2.27 (0.55), Efficiency 1.73 (0.52), Dependability 1.50 (0.91), Stimulation 1.50 (0.76), and Novelty 1.05 (0.97) [Table 1]. Pragmatic Quality (the mean of Perspicuity, Efficiency, and Dependability) was 1.83 and Hedonic Quality (Stimulation and Novelty) 1.27. Compared with the published UEQ benchmark dataset, scores ranged from above-average to excellent, with Perspicuity and overall pragmatic quality in the highest bands, indicating that users found the simulator clear, efficient, and dependable. The comparatively lower Novelty score and the wider dispersion on Dependability and Novelty, driven largely by a single more critical respondent, are consistent with a tool that prioritizes clinical utility over novelty. These data are reported transparently and descriptively given the small convenience sample (*n* = 10) coinciding with the development cohort, the single assessment time point, and the absence of a comparator.

**Table 1:** User Experience Questionnaire (UEQ) results for Open Vent Sim at the end of iterative development (*n* = 10; seven residents and three attending physicians). Values are scale means on the − 3 to + 3 metric, with standard deviations and 95% confidence intervals

Scale	Mean (− 3 to + 3)	SD	95% CI
Attractiveness	1.68	0.73	1.23 to 2.14
Perspicuity	2.27	0.55	1.94 to 2.61
Efficiency	1.73	0.52	1.40 to 2.05
Dependability	1.50	0.91	0.94 to 2.06
Stimulation	1.50	0.76	1.03 to 1.97
Novelty	1.05	0.97	0.45 to 1.65

## Discussion

### Educational impact and resource optimization

The implementation across SimZone 1 and SimZone 2 demonstrated practical solutions to common resource constraints in simulation-based education. By replacing unavailable anesthesia machines and expanding beyond single-user commercial simulators, the application addressed specific institutional needs while maintaining educational effectiveness. The multi-user capability and scenario flexibility enable diverse training applications, from basic resident education to specialized procedure training, making custom-developed solutions a key advantage over commercial alternatives with fixed functionality.

### Technical development effort and resource requirements

The NLDD approach significantly reduced the initial development barrier, enabling clinical experts to rapidly create a functional prototype. However, the transition from prototype to production-ready application required additional technical effort. The 40 h of clinical team development time were supplemented by technical specialist work focused on code refactoring, architectural improvements, and quality assurance. A structured approach to resources needed is presented in Table 2.

**Table 2:** Human and technical resources required to implement a Natural Language-Driven Development (NLDD) project in healthcare simulation. For each resource, the table indicates the level of necessity (Required, Optional, or Minimal), key requirements or competencies, and representative examples or options.

Resource Category	Essential Requirements	Details	Examples/Options
HUMAN RESOURCES			
Clinical Domain Expert	Required	• Clear problem definition • Subject-matter expertise • Guide simulation content • Ultimate authority for clinical accuracy	• Clinician educator • Primary designer (not support role)
Technical Facilitator	Required	• Web development concepts • Version control systems • API interaction & client-server architecture • Debugging tools & error message interpretation • Cloud deployment experience • Learning adaptability • Translation between clinical/technical requirements	• Set up development environment • Help phrase technical prompts • Manage code versions/backups • Identify refactoring needs • Coordinate deployment
Additional Team Members	Optional	• Additional clinicians for broader input • Tech support for complex programming/UI design • Experienced developers for code review	• Larger projects only • Periodic architecture reviews
TECHNICAL RESOURCES			
Development Platform	Required	Cloud-based AI coding environment	
AI Model	Required	Large Language Model with coding capabilities	
Version Control	Required	Code management and collaboration	
Deployment Platform	Required	Hosting for web application	
Hardware	Minimal	Computer with internet access	
Software Costs	Minimal	Virtually zero through free services	

### Human resources

The minimal team for an NLDD project should include: (1) a Clinical Domain Expert – a clinician educator with clear problem definition and subject-matter expertise to guide the simulation’s content – and (2) a Technical Facilitator. The technical facilitator’s role requires: general understanding of web development concepts (HTML, CSS, JavaScript basics), familiarity with version control systems (particularly Git), ability to interact with Application Programming Interfaces (APIs) and understand basic client-server architecture, comfort with debugging tools and reading error messages, and experience with deploying web applications to cloud services.

Importantly, deep expertise in specific frameworks like React is not essential from the start – the AI can handle framework-specific syntax. The facilitator should be comfortable learning new tools as needed and translating between clinical requirements and technical possibilities. Their primary responsibilities include setting up the development environment, helping phrase technical prompts when the AI misunderstands clinical intent, managing code versions and backups, identifying when code refactoring is needed for maintainability, and coordinating the deployment process. While larger projects may benefit from additional clinicians for broader input or tech support for complex programming, the core principle remains: clinicians are the primary designers with tech personnel supporting that process.

### Cost estimation

Hardware requirements are limited to internet-connected computers, while software costs approach zero through freely available services, making NLDD highly accessible even to resource-constrained institutions.

The primary investment is the clinical lead’s time to iteratively develop the tool. In our experience, initial prototypes can be created within days to weeks, but achieving polished, fully-featured simulators may require several iterative cycles over months. The clinician should budget dedicated time for this creative process, akin to writing a curriculum or scholarly projects. Some AI tool familiarization is useful upfront – understanding effective prompting, interpreting AI code suggestions, and result verification. After an initial learning curve, clinicians become adept at “communicating” with AI to yield desired outcomes. While basic programming framework understanding (e.g., React’s component structure, variable, and function concepts) can accelerate progress, it is not strictly required to start.

### Potential pitfalls and workarounds

#### Code Maintainability

AI-generated code can sometimes be inefficient. The technical facilitator should regularly review code for clarity. As projects grow, periodic reviews of the software architecture by experienced developers are advisable.

#### The “Black Box” problem

The AI’s reasoning is not always transparent, but the Gemini LLM “thinks out loud” in the chat panel, allowing clinicians to follow reasoning and understand code generation while enabling mistake correction. When AI makes errors, provide clear, specific, and simple corrective feedback.

#### Over-reliance on the AI

The AI is a tool, not a creator. It cannot validate clinical accuracy. The clinical expert must remain the ultimate authority, rigorously testing and reviewing every feature against real-world knowledge.

### Lessons learned

The development of a mechanical ventilator simulator using clinician-centered NLDD methodology established a working framework generalizable to other specialties and educational needs. Analysis of development phases addressed important issues, ensuring project feasibility.

#### Start with clear, specific problems and a well-structured design

 Our project succeeded because it addressed a specific need, the lack of ventilator training solutions under resource constraints. Successful NLDD requires comprehensive upfront design work: clearly defining problems, educational objectives, target user workflows, and core functional requirements before any AI prompting begins.

#### Rapid iteration 

NLDD leverages AI strength in generating quick outputs, making “try it and see” approaches effective. [18] Iteration means being prepared for failures – when AI produces incorrect features, re-prompt more clearly or break tasks down into smaller steps. After reaching certain milestones, having developers or technical facilitators optimize the whole project (performance, security) proves beneficial.

#### The technical facilitator role is crucial

 This person bridges clinicians’ vision and the realities of technical code. We advocate training more clinicians in basic coding literacy and simulation technicians in the effective use of AI coding tools, thereby smoothing future collaborations.

### Healthcare simulation applications

The potential application of NLDD in healthcare simulation is vast. Any educator with clear ideas for custom tools can use this approach to create functional prototypes with minimal resources. It is particularly well-suited for rapid curriculum development and promoting open-source culture in medical technology.

#### Limitations and future directions

While this work demonstrates, with tangible examples, how NLDD promotes clinician-made, customized digital simulators, this initial study was conducted at two centers with a single team focused on a specific medical specialty. A key limitation of the current study is the absence of a formal quantitative assessment of pedagogical outcomes. Usability and user experience were assessed with a validated instrument (the UEQ), but no validated learning-outcome measures or pre-post knowledge assessments were employed; moreover, the UEQ was completed by a small convenience sample (*n* = 10) coinciding 

with the development cohort, at a single time point and without a comparator. This choice was made to focus on the simulator’s design feasibility and optimization; however, it limits the strength of conclusions that can be drawn about the simulator’s training value. The present study therefore establishes the tool’s fidelity and usability, whereas its educational effectiveness remains to be demonstrated. Future studies should incorporate validated knowledge assessments, structured objective measures of ventilation competence, and comparative evaluations against existing training modalities to establish the educational effectiveness of NLDD-developed simulators. While feedback was positive, a formal multicentre evaluation represents the necessary next step. Additional studies on the use of NLDD-made simulators are required.

Long-term durability and scalability of applications built entirely this way remain undetermined. Questions regarding optimal clinician training for AI prompting, intellectual property handling when AI co-develops code, and ethical considerations (ensuring AI does not introduce errors, bias, or unsafe suggestions) require further work. Not every clinician will be comfortable working with AI in this manner, and organizational support and training will be required to encourage NLDD adoption in simulation centers.

## Conclusion

Natural Language-Driven Development represents a significant shift in the creation of educational technology. By empowering clinical experts to become designers of their own tools, it closes the clinician-developer gap and places the power of innovation directly in the hands of medical education frontline practitioners. The successful creation of Open Vent Sim, a mechanical ventilator simulator, validates this approach’s generalizability to other domains.

Transforming prototypes into robust, maintainable products requires significant additional development effort. [29, 30] This duality demonstrates that with appropriate AI tools and clear educational needs, any educator can move ideas from simple prompts to practical, customized simulations for learners, though professional technical guidance remains essential for production-ready systems.

Importantly, NLDD does not eliminate technical expertise requirements but rather redefines collaboration between different professionals involved in healthcare simulation.

This evolution places greater emphasis on upfront design, project planning, and the definition of control criteria, with AI handling subsequent implementation work. AI is not a replacement for human expertise but a tool that amplifies it, enabling clinical educators to transform domain knowledge directly into educational innovations that serve learners’ needs.

## Supplementary Information

Supplementary Material 1.

## Data Availability

The source code for *Open Vent Sim* is available on GitHub (https://github.com/fedebarra/open-vent-sim/).

## Abbreviations

AI: Artificial Intelligence
ARDS: Acute Respiratory Distress Syndrome
COPD: Chronic Obstructive Pulmonary Disease
ICU: Intensive Care Unit
LLM: Large Language Model
NLDD: Natural Language-Driven Development
UI: User Interface
